# Understanding the development of bacterial colony: Physiology, new technology, and modeling

**DOI:** 10.1002/qub2.95

**Published:** 2025-04-17

**Authors:** Jingwen Zhu, Pan Chu, Xiongfei Fu

**Affiliations:** ^1^ State Key Laboratory for Quantitative Synthetic Biology Shenzhen Institute of Synthetic Biology Shenzhen Institutes of Advanced Technology Chinese Academy of Sciences Shenzhen China; ^2^ University of Chinese Academy of Sciences Beijing China

**Keywords:** bacterial colony, mathematical model, physiological interaction, quantitative technology

## Abstract

Bacterial colonies, as dynamic ecosystems, display intricate behaviors and organizational structures that profoundly influence their survival and functionality. These communities engage in physiological and social interactions, resulting in remarkable spatial heterogeneity. Recent advancements in technology and modeling have significantly enhanced our comprehension of these phenomena, shedding light on the underlying mechanisms governing bacterial colony development. In this review, we explore the multifaceted aspects of bacterial colonies, emphasizing their physiological intricacies, innovative research tools, and predictive modeling approaches. By integrating diverse perspectives, we aim to deepen our understanding of these microbial communities and pave the way for novel applications in biotechnology, ecology, and medicine.

## 1 INTRODUCTION

Bacterial colonies, whether thriving on surfaces, within biofilms, or in natural environments, constitute intricate microcosms where individual cells collaborate, compete, and adapt. These colonies exhibit complex behaviors and organizational structures that profoundly impact their survival and functionality. Contrary to the outdated view of bacteria as mere “single‐celled organisms,” bacterial colonies or biofilms display behaviors and characteristics akin to those of multicellular organisms [1, 2]. They possess intricate communication capabilities, sophisticated coordinated behaviors, and adaptive benefits derived from multicellular cooperation and competition [3, 4, 5, 6, 7, 8]. These include direct cell‐to‐cell and cell‐to‐surface physical interactions [9, 10, 11, 12, 13, 14], indirect physical interactions (e.g., swarming [15, 16, 17]), long‐range signaling (e.g., quorum sensing [18, 19]), chemotactic signaling [20, 21, 22, 23, 24], collective activation and deactivation of genes [25], and even the exchange of genes [26, 27].

Additionally, individual bacteria possess complex signal transduction and gene regulatory networks that integrate signals, such as microenvironment cues and morphogen gradients, combined with stochastic gene expression events, to regulate cellular differentiation and cell fate specification. Leveraging these capabilities, as multicellular communities, colonies develop intricate spatiotemporal patterns in response to adverse conditions, thereby enhancing their resilience and adaptability.

Understanding how these microbial communities evolve, organize, and spatially structure themselves through quantitative measurements and modeling is crucial for managing microorganism‐related challenges in medical, industrial, and environmental contexts. This knowledge also holds immense value for biotechnological applications, leading to innovative practical solutions. Furthermore, unraveling the pattern formation of living communities and their hidden mechanisms serves as a cornerstone in the fields of developmental biology [28, 29, 30], ecology, and evolution.

In this review, we explore the multifaceted aspects of bacterial colonies as multicellular entities, emphasizing their physiological intricacies and the social interactions within communities and their environments. Additionally, we highlight innovative quantitative measurement tools and predictive modeling approaches employed to study the development of bacterial colonies.

## 2 PHYSIOLOGICAL INTERACTIONS BETWEEN BACTERIAL COLONIES AND ENVIRONMENT

### 2.1 Heterogeneity in colonies

The structure and chemical conditions within bacterial colonies can vary both spatially and temporally, leading to physiological heterogeneity. This heterogeneity may manifest as differences in metabolic activity or growth states among cells. Gradients of nutrients, oxygen, metabolites, and waste products naturally arise due to cellular metabolism and spatial variations, creating distinct microenvironments. These microenvironments contribute to the emergence of subpopulations within the colony, each with unique gene expression profiles, as previously reviewed [31, 32, 33, 34, 35].

**FIGURE 1:**
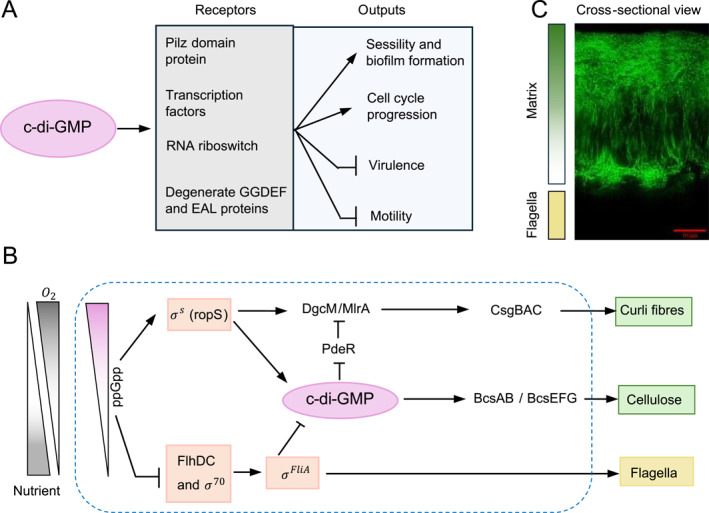
Graphic overview of global regulatory mechanisms in *Escherichia coli* colony biofilm formation. (A) Illustration of the impact of cyclic di‐GMP (c‐di‐GMP), highlighting its main receptors and downstream regulatory effects. (B) Regulatory networks influencing *E. coli* colony architecture encompass environmental resource gradients (oxygen and nutrients), guanosine pentaphosphate (ppGpp), sigma factors, and c‐di‐GMP levels. Intracellular c‐di‐GMP signaling promotes cellulose production, a primary biofilm matrix component, while $\sigma^{s}$ (RpoS) enhances curli fiber synthesis, strengthening the matrix structure. The transcriptional regulator FlhDC controls the expression of sigma factor FliA, which promotes flagella formation and assembly. The global regulator ppGpp, responsive to resource gradients, further modulates these pathways, balancing biofilm matrix production and motility. (C) Cross‐sectional view of an *E. coli* K‐12 colony biofilm, illustrating matrix production in the upper and intermediate sections of the colony, with a mesh‐like flagellar structure at the biofilm base (not shown).

Global regulations, such as c‐di‐GMP signaling [36, 37], the spatial organization of differential *σ* factor activities, and the stringent control of ribosomal gene expression, play crucial roles in determining the physiological states of subpopulations within bacterial colonies (Figure 1). The spatial interplay between these global gene regulation mechanisms and microenvironmental conditions within colonies underscores the complexity of bacterial community dynamics. This intricate regulation ensures that different cell types within the colony can specialize and adapt to their specific microenvironments, contributing to the overall resilience and functionality of the bacterial community.

When considering the global regulation of cell physiology, cells in a specific growth state exhibit properties that tend to correlate, regardless of whether that state is induced by conditions in a homogeneous liquid culture or within a colony. This correlation arises because these properties are driven by overarching shifts in gene expression that are common across different environments. For example, *Bacillus subtilis* forms three distinct cell types—matrix‐producing, motile, and sporulating—each defined by a unique genetic program reflecting dynamic changes in the colony’s physiological landscape [38]. In a microcolony biofilm, motile cells are localized at the bottom, sporulating cells at the top, and matrix‐producing cells span the middle region of the biofilm. These distributions are thought to result from pathways triggered by microenvironmental conditions, mirroring those in liquid culture. This spatial organization of cell types within the biofilm highlights the influence of localized environmental factors on gene expression and cellular differentiation [39, 40, 41, 42, 43, 44], showcasing the intricate regulation that governs colony development.

However, some emergent properties within colonies cannot be observed in homogeneous liquid conditions, regardless of whether the environment is balanced or altered. When viewed as multicellular communities, bacterial colonies exhibit complex social interactions that give rise to diverse phenomena. These interactions within colonies can lead to behaviors and properties—such as collective resource management, cooperative defense mechanisms, and intricate spatial organization—that are absent or less pronounced in liquid cultures. These emergent properties are a direct consequence of the structured, spatially heterogeneous environments within colonies, where close cell‐to‐cell communication and environmental gradients drive unique adaptive strategies.

### 2.2 Social interactions within colonies

#### 2.2.1 Intercellular communication

**FIGURE 2:**
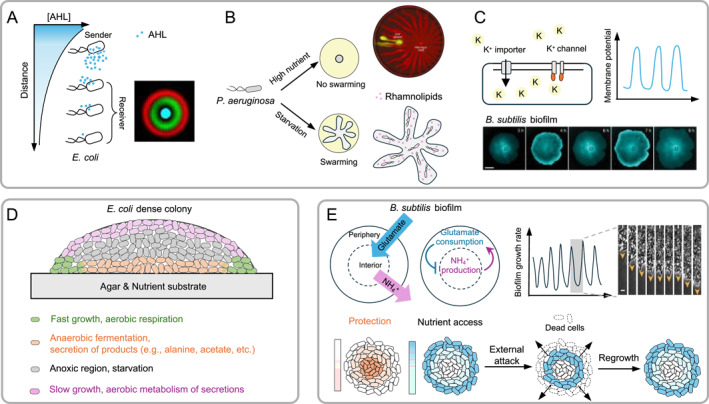
Social interactions and consequences of heterogeneity in bacterial colonies. (A–C) intercellular communication mechanisms, (D) metabolic cross‐feeding, and (E) cooperation and competition behaviors in bacterial communities. (A) A synthetic gene network in *Escherichia coli* utilizing quorum sensing of the chemical signal acyl‐homoserine lactone (AHL) facilitates intercellular communication, resulting in the differentiation of cells to form a bullseye pattern. Figure reproduced with permission from Basu et al. [18]. (B) Cooperative behavior in *Pseudomonas aeruginosa* colony formation, where the production of rhamnolipids—a biosurfactant—regulates swarming and enables the formation of a branched colony under nutrient‐starvation conditions. (C) Long‐range electrical signaling via potassium ion channels induces collective oscillations of membrane potential in *Bacillus subtilis* biofilms, balancing competing metabolic demands within the colony. Figure reproduced with permission from Prindle et al. [45]. (D) Metabolic cross‐feeding within a dense bacterial colony leads to spatial division of metabolic processes, allowing subpopulations to specialize based on their location. (E) In a *B. subtilis* biofilm, the peripheral cells consume nutrients, which leads to the starvation of the interior cells. However, these peripheral cells also shield the interior cells from external dangers. This dilemma between nutrient deprivation and protection is resolved through the development of long‐range metabolic communication among the peripheral and interior cells. Figure reproduced with permission from Liu et al. [46].

Bacteria exhibit sophisticated intercellular communication, with much of the research traditionally focusing on chemical signaling systems, particularly quorum sensing. This process involves the release of signaling molecules, whose accumulation is driven by increasing population density. The molecules are detected by neighboring cells, enabling collective behaviors within the community. A notable example is the work of Basu et al. [18], who engineered a synthetic gene network that integrates quorum sensing, enabling cells to communicate and differentiate based on external signals [18]. This system facilitated the programmed formation of ring‐like differentiation patterns within a bacterial colony, driven by chemical gradients of acyl‐homoserine lactone signals (Figure 2A). The synthetic network effectively utilized diffusible signals to relay information between cells, leading to coordinated behavior and pattern formation.

In *Pseudomonas aeruginosa*, quorum sensing also plays a crucial role in colony formation by regulating the production of rhamnolipids—glycolipid biosurfactants essential for biofilm development and maintenance [17, 47, 48]. Rhamnolipids reduce surface tension, enabling the bacteria to spread more effectively across surfaces, particularly under nutrient‐starved conditions [49]. They also promote bacterial aggregation, which facilitates the formation of robust biofilm structures. The *rhl* gene cluster, responsible for rhamnolipid production, is regulated through quorum sensing, ensuring that these biosurfactants are produced at levels optimized for survival and virulence in fluctuating environmental conditions. Additionally, *P. aeruginosa* communities exhibit cooperative behavior, where cells lacking the *rhlA* synthesis gene can swarm by utilizing the rhamnolipids produced by other cells (Figure 2B).

Beyond chemical signaling, emerging evidence suggests that some bacteria may also utilize electrical signals to coordinate activities across populations. In *B. subtilis* biofilms, cells at the periphery can send long‐range electrical signals to those at the center, resulting in collective oscillations that help balance competing metabolic demands within the biofilm (Figure 2C). These electrical signals are transmitted through potassium ion channels embedded in the biofilm matrix, which act as bridges between closely positioned cells, ensuring efficient electrical communications through electron flux across the biofilm [45].

#### 2.2.2 Division of labor

The complex metabolic network within bacterial colonies plays a crucial role in social interactions, particularly through the coordinated division of labor among subpopulations. Cross‐feeding is one such division of labor, where distinct subpopulations specialize in different metabolic processes, benefiting from the activities of their neighbors. This optimization of resource use maximizes the growth or survival of the entire colony.

Recent studies in *Escherichia coli* colonies have shown that alanine metabolism is both spatially and temporally heterogeneous, significantly affecting local growth dynamics. Díaz‐Pascual et al. [50] demonstrated the existence of a metabolic interplay between the anoxic base of the colony, near the nutritive agar where alanine is secreted, and the oxygen‐rich, nutrient‐deprived top of the colony, where alanine is subsequently utilized as a carbon source [50]. This spatial division of metabolic processes results in the specialization of subpopulations based on their location within the colony, shaping the overall architecture and function of the colony (Figure 2D). Similar metabolic cross‐feeding phenomena [51] have been observed for other compounds, such as acetate [52, 53, 54], amino acids [50, 55], and nitrogen compounds [56]. These metabolic exchanges create diverse selection pressures at different points throughout the developing community, leading to differential gene expression and physiological heterogeneity within the colony.

Liu et al. [46] demonstrated how metabolic activity within *B. subtilis* biofilms is organized both spatially and temporally, leading to a codependence between interior and peripheral cells [46]. This codependence results in collective oscillations that enhance the biofilm’s resilience, allowing it to grow larger while maintaining viable interior cells. Manipulating these oscillations could offer strategies to control biofilm growth, potentially making biofilms more vulnerable by promoting continuous peripheral growth and starving the interior. This dynamic interaction effectively mitigates the social conflict between cooperation (protection) and competition (starvation), balancing the overall needs of the biofilm community (Figure 2E).

## 3 QUANTITATIVE MEASUREMENT TECHNIQUES

Intricate physiological processes and spatial organizations dictate the functionality and resilience of bacterial colonies. To unravel these complexities, researchers have developed and refined a diverse array of quantitative techniques. Recent technological advances in understanding the physiology, genetics, and morphology of microorganisms growing in colonies offer unprecedented insights into their structural, functional, micro‐gradient, and metabolic dynamics [57]. These techniques, ranging from molecular analysis tools and advanced imaging to next‐generation sequencing technologies and microfluidic approaches, play a pivotal role in elucidating the complexities of microbial communities.

Since the 1980s, significant advancements have been made in imaging techniques for bacterial colonies [58, 59]. With the integration of development of microscopy techniques, fluorescence‐associated tools, genetic engineering, computing, and artificial intelligence for image processing [57, 60], it provides an unprecedented opportunity to deepen our understanding of bacterial biofilms and colonies.

### 3.1 Fluorescence‐based microscopy

Wide‐field microscopy and laser scanning microscopy are widely employed to observe bacterial cells or communities, as detailed in Table 1. Among these, confocal laser scanning microscopy (CLSM) has significantly advanced research on bacterial colonies and biofilms due to their three‐dimensional structures. Lawrence and coworkers [69] were pioneers in using CLSM to obtain three‐dimensional images of biofilms. Since then, CLSM has become a fundamental tool in almost all studies of colony or biofilm structure and development [64, 70, 71]. CLSM is ideal for imaging biofilms because it effectively captures their extensive three‐dimensional structures, which can be stained with fluorophores [72, 73], fluorescent probes (e.g., fluorescent in situ hybridization [74, 75, 76] and organic dyes [77]), and fluorescent proteins [61, 78]. CLSM systems offer high‐resolution, high‐contrast imaging for biological biofilms. However, the slow acquisition of large images and the risk of photobleaching and photodamage due to prolonged exposure to high‐intensity laser light are limitations.

**TABLE 1:** Imaging techniques for bacterial colonies.

	Advantages	Limits	Applications	References
Wide‐field microscopy	Faster imaging speeds than high‐resolution microcopy	Low resolution	Acquisition images of colony pattern and a 2D colony in microfluidic devices with fluorescent labels	[61, 62]
	Simplicity and low cost	Low optical sectioning ability and only getting the colony bottom image		
Confocal laser scanning microscopy (CLSM)	High‐resolution imaging of bacterial colonies	Low axial resolution	Acquisition high‐resolution images of colony patterns and 2D colonies in microfluidic devices with fluorescent labels	[63, 64]
		Restricted penetration depth		
		Limit imaging speed for capturing rapid changes in real time		
		High light intensity focused on a single point may cause photobleaching and phototoxicity		
Spinning‐disk confocal scanning microscopy (compared to CLSM)	Faster imaging speeds	Lower axial (*z*‐axis) resolution	Live‐cell and long‐term imaging	[14, 65]
	Suitable for live‐cell imaging and dynamic processes	Lower light transmission		
		Fixed pinhole and slightly lower resolution		
Two/multiphoton laser scanning microscopy	Higher penetration of IR light into the sample	Slightly lower resolution than the single‐photon microscopy	Imaging structure inside dense colonies	[54, 66]
	Imaging thick samples allows imaging up to 1 mm deep in biological tissues			
	Reduced photodamage and photobleaching, beneficial for live‐cell and long‐term imaging	Fluorophore limitations		
Light‐sheet fluorescence microscopy (LSFM)	Minimal photodamage and photobleaching penetrate deeper into thick samples with minimal light scattering and absorption and high‐speed image acquisition	Sample preparation: optically cleared samples are needed	Imaging of large biological specimens such as embryos, organoids, and entire small organisms	[67, 68]
			Capture fast biological processes and dynamics in living specimens	

To address these limitations, spinning‐disk confocal scanning microscopy was developed [79, 80, 81]. This technique uses a rapidly rotating disk covered with an array of microlenses and pinholes to scan the sample, allowing simultaneous illumination of multiple points and reducing out‐of‐focus light. This method is particularly advantageous for live‐cell imaging due to its ability to capture dynamic processes with minimal photodamage and photobleaching, such as the dynamics of cell alignment, orientation, and initial architecture of bacterial colonies [82, 83, 84]. In addition, two‐photon or multiphoton microscopy offers superior depth penetration compared to single‐photon laser scanning microscopy because the near‐infrared (NIR) laser scatters less in biological tissues. This technique also reduces photobleaching and photodamage, as excitation is confined to the focal point, and the NIR light is less damaging to biological tissues. These features make two‐photon microscopy ideal for deep tissue and live imaging. Recent studies have demonstrated significant benefits for imaging fluorescence distributions within thicker bacterial colonies [54, 66].

In recent years, light‐sheet fluorescence microscopy (LSFM) has emerged as a powerful imaging technique for studying three‐dimensional structures. LSFM enables researchers to visualize biofilm architecture in real time, capturing dynamic processes such as growth, division, and interactions within colonies [85, 86]. By illuminating specimens with a thin sheet of laser light, this method reduces phototoxicity and photobleaching while providing excellent 3D spatial resolution and fast temporal resolution, whose levels are unmatched by confocal microscopy. For example, Qin et al. used light‐sheet microscopy to study cell position fates and collective fountain flow phenomena in *Vibrio cholerae* biofilms, revealing dynamic behaviors and structures within bacterial biofilms [67]. LSFM also facilitates accurate cell detection and cellular shape measurement in densely packed 3D biofilms of *E. coli* and *Myxococcus xanthus* [68, 87].

Additionally, these imaging techniques can be coupled with other advanced methods to perform spatially resolved chemical information imaging. Techniques such as Fourier‐transform infrared spectroscopy [88, 89, 90], matrix‐assisted laser desorption/ionization mass spectrometry imaging [91, 92, 93], and Raman spectroscopy [94, 95] allow for the analysis of biochemical signals within both cells and extracellular substances. These methods reveal how biochemistry changes as a function of growth conditions, providing deeper insights into the microenvironment of colonies.

### 3.2 Workflows for spatial–temporal analysis of bacterial colonies

Historically, the absence of methods capable of profiling genomewide activities with spatial resolution has hindered our understanding of spatially specific properties within bacterial colonies, often limiting studies to a case‐by‐case basis [96, 97, 98]. However, recent advancements have revolutionized our ability to visualize subpopulations and gradients (e.g., chemical and signaling molecules) within colony structures.

**FIGURE 3:**
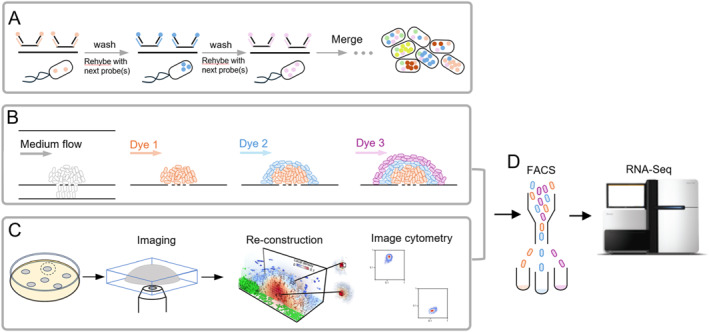
Experimental workflows for studying gene expression in bacterial colonies using fluorescence labeling. (A) par‐seqFISH (parallel sequential fluorescence in situ hybridization), a high‐throughput technique that captures gene expression profiles of individual bacteria within a biofilm, maintaining the spatial organization and physical context within structured environments. (B) RAINBOW‐seq, a spatial transcriptomics approach that integrates growth‐based encoding with fluorescence‐activated cell sorting (FACS, shown in panel D) to profile the transcriptome of *Escherichia coli* biofilm communities, achieving high spatial resolution and comprehensive gene coverage. (C) Image cytometry, a method developed for the quantification, analysis, and visualization of fluorescent protein expression distribution in bacterial colonies, providing insights into their three‐dimensional spatial and temporal dynamics.

For example, using *P. aeruginosa* as a model, Dar et al. [75] developed the parallel sequential fluorescence in situ hybridization (par‐seqFISH) technique. This innovative method allowed for the study of spatial gene expression profiles at the single‐cell level, specifically analyzing 105 genes associated with cell physiology across more than 600,000 *P. aeruginosa* biofilm cells (Figure 3A). Their research uncovered heterogeneous expression patterns related to pyocins, flagella, and type IV pili production throughout the biofilm. Additionally, transcripts involved in anaerobic respiration were found to be localized within specific microenvironments deeper inside the biofilm. This study highlights the existence of distinct subpopulations within a single biofilm, each with unique gene expression profiles and physiological states.

Flow cytometry serves as a robust tool for analyzing individual cells within complex populations, providing quantitative data on cellular parameters with high sensitivity and throughput. This technique is crucial for understanding colony dynamics by assessing heterogeneity within bacterial populations. By measuring fluorescence and light scattering properties, flow cytometry allows for the high‐throughput characterization of cell viability, size, complexity, and physiological states within bacterial colonies. Fluorescence‐activated cell sorting (FACS) further enhances this capability by isolating specific subpopulations based on fluorescence profiles, facilitating downstream molecular analyses such as spatially resolved transcriptomics and metabolomics [50, 66, 77]. However, flow cytometry lacks spatial resolution and cannot capture the spatial organization of cells within biofilms or colonies. To address this limitation, integrating flow cytometry with advanced microscopy techniques could enhance its spatial resolution. Wang et al. introduced a novel method named RAINBOW‐Seq [77], which employs a growth‐based encoding strategy to label biofilms in a spatially specific manner, enabling the analysis of the transcriptome in *E. coli* biofilm communities (Figure 3B). This approach allowed the identification of several modes of community‐level metabolic coordination, including cross‐regional resource allocation, local cycling, and feedback signaling.

In another recent work [66], the authors employed a two‐photon microscope to dissect the spatial structures within dense *E. coli* colonies harboring by a bistable genetic circuit [66]. They reconstructed the spatial distribution of fluorescence intensity using the optical section acquired from two‐photon microscopy and subsequently sorted bacterial cells based on their fluorescence intensities in the colony using FACS (Figure 3C).

Tools like BiofilmQ [99] further enhance our ability to analyze spatial and temporal aspects of fluorescence, cytometry, and community architecture within biofilms. BiofilmQ supports cube segmentation and the import of single‐cell segmentation data from fluorescence imaging, facilitating global measurements of microbial communities and advanced data visualization (Figure 3C).

Collectively, these techniques are driving advancements in our understanding of bacterial colony development. By integrating these methodologies, researchers are poised to uncover new insights into microbial ecology, biotechnological applications, and medical interventions. Each technique contributes unique strengths, collectively enhancing our ability to decode the complexities of microbial communities and their interactions with their environments.

### 3.3 Other techniques

In addition to commonly used microscopy and imaging methods, several advanced techniques offer quantitative insights into bacterial colony morphology and gene expression at high spatial resolution. Electron microscopy, including scanning electron microscopy and transmission electron microscopy, provides detailed images of colony surface topography and internal structures at nanometer‐scale resolution. These techniques are particularly valuable for visualizing extracellular matrix components and the organization of bacterial cells within biofilms. Atomic force microscopy (AFM) offers a complementary approach by enabling three‐dimensional surface mapping of colonies with minimal sample preparation. AFM provides precise measurements of cellular and matrix features at the nanoscale and can quantify mechanical properties, such as cell stiffness and biofilm surface roughness, adding a layer of functional analysis to morphological studies. For assessing gene expression within colonies, mRNA fluorescence in situ hybridization allows for spatial localization and quantification of specific transcripts within individual cells. This method enables researchers to study gene expression patterns in relation to colony structure and local environmental conditions. Reverse transcription quantitative PCR is another powerful technique for quantifying gene expression levels, providing a sensitive measure of gene transcription activity across different regions of a colony or in response to environmental changes.

Together, these methods offer a comprehensive tool kit for studying the spatial and functional organization of bacterial colonies, with recent reviews providing further insights into their applications and technical considerations [42, 100, 101].

## 4 MODELING OF BACTERIAL COLONIES

The formation of bacterial colonies is a complex process that goes beyond mere cell reproduction. Numerous studies have provided valuable insights into the multifaceted mechanisms involved. Colony formation is influenced by various factors, including growth, division, metabolism, nutrient diffusion, bacterial movement, mechanical interactions, reproduction, and local communication. While the morphology of bacterial colonies can be experimentally observed and controlled, identifying the key drivers and fully understanding the underlying mechanisms remain challenging, as most processes during colony formation are nonlinear and dynamic. Additionally, it is impossible to independently control certain cellular parameters, such as growth rate, cells size, and motility, in experiments [102]. To understand the roles of these parameters in these nonlinear systems, mathematical modeling provides a promising strategy.

Over the past few decades, numerous mathematical models have been developed to describe bacterial colony growth and pattern formation. Most of these models are based on the reaction–diffusion equation [15, 103, 104, 105, 106, 107, 108]. These models integrate bacterial diffusion, growth, and nutrient diffusion to simulate colony expansion. In this framework, cell density is characterized in a continuous field, and cell swimming and diffusion are characterized by diffusion terms. However, these models have limitations when applied to dense bacterial colonies, where cells are often packed and trapped by the extracellular matrix, and migration is dependent on mechanical interactions. For example, cell migration is often impeded by factors such as cell adhesion to surfaces and frictional forces. To overcome these limitations, continuum models that treat the colony as growing fluids or viscoelastic materials have been developed [67, 109, 110, 111, 112]. At a macroscopic scale, these models incorporate microscopic factors—such as cell elongation, division, and the continuous production of the extracellular matrix—as a small number of hypothetical variables, which collectively contribute to the cohesive expansion of the colony. Furthermore, by introducing order parameters, it becomes possible to study systems with multiple species or examine the system at the microscopic scale. For example, Xiong et al. [112] introduced the phase field into this framework, enabling the study of patterns emerging from interactions between two strains with different motilities [112]. To investigate cell‐to‐cell interactions, researchers have employed frameworks from the study of dense liquid crystals. By introducing pseudovectors to represent individual cells, it becomes possible to study how cell ordering is aligned by cell growth and colony expansion [109].

However, this approach does not fully capture the growth dynamics on surfaces at a microscopic level and neglects many details in mechanics and cell‐to‐cell heterogeneities. For example, expansion is frequently driven by cells physically displacing each other as they grow, rather than by migration alone. Additionally, cell growth and division are not synchronized, and phenotypes fluctuate.

To address these limitations, individual‐based models (IBMs) or agent‐based models (ABMs) have been developed [54, 64, 82, 113]. These models incorporate mechanical interactions in the growth of dense colonies on solid substrates, offering a more detailed representation of the physical interactions within colonies. Pioneering work by Warren et al. [64] quantified the forces and key elements that influence the spatiotemporal development of bacterial colonies on hard agar, highlighting the crucial role of surface tension in radial cell expansion.

However, these models often lack the integration of biochemical signaling, which plays a crucial role in colony formation. While IBMs/ABMs provide a closer approximation of the physical dynamics, they may still fall short in capturing the complex interplay between biochemical and mechanical factors that drive colony development. Additionally, these models can be computationally expensive, limiting their practicality for simulating large‐scale or long‐term colony growth. In reality, a typical colony that develops for 12 h may contain more than 10^8^ cells, making it computationally infeasible to model such a large number of cells on a standard workstation at present.

Cell automaton (CA) models more closely resemble IBMs but are computationally less demanding at similar scales. This is because they approximate cell division, migration, growth, and cell‐to‐cell interaction to simple rules. Moreover, these models offer greater flexibility in terms of scales in simulations; lattice sites can represent either a single cell or a small territory of cells of one species. Ben‐Jacob et al. [104] provided an impressive example of using this generic framework to explore the colony pattern formations under different nutrient conditions and agar concentrations. Based on simple rules, their work revealed how nutrient and migration limitations profoundly influence branch formation during colony expansion. However, a drawback of this framework is that the rules may be too abstract to dissect the mechanism details. Additionally, patterns simulated by CA frameworks may tend to elongate along lattice axes, resulting in anisotropic patterns [114]. Researchers should be aware of the need to use appropriate lattice types, and off‐lattice models are also optional methods to overcome this issue.

During colony development, the system starts with a few cells and grows into a dense ensemble of billions within millimeters. This process spans multiple scales in both time and space, causing significant challenges in balancing computational load and accuracy. Analyzing scale changes during simulations is crucial, and selecting an appropriate framework—or using a hybrid approach—is essential for investigating mechanisms of interest. Here, we summarized these types of models in Table 2.

**TABLE 2:** Table for comparing different models.

Model	Expansion mechanism	Context of use
Continuum mechanics	Fluid and viscoelastic mechanics	* Biofilm wrinkling [111] * Swarming system and pattern formation [15, 103, 104, 105, 115] * Colony growth on the solid surface [116] * Branched pattern of colonies [16] * Flower‐like pattern and cell‐to‐cell interaction [112]
	Cell diffusion and swimming	
	Phenomenological	
Discrete‐element model (IBMs and ABMs)	Granular‐level forces	* Cell ordering in single‐layer bacterial community [109] * Branched colony formation [10] * Dense colony establishment [64]
Cellular automation	Non‐Newtonian mechanics (rule‐based expansion)	* Branched colony formation [104] * Cross‐feeding and metabolic interactions [117] * Cross‐feeding and spatial organization [118]

To study patterns emerging from different cell strains or heterogeneities among cells, most research utilizes IBM frameworks. Simulating cells with discrete elements allows for precise representation of cell properties. Introducing variations in gene expression, growth, and cell division is straightforward and has been successfully applied in various scenarios, including species segmentation, competition, genetic drift, and cell fate determination. However, when simulations include nutrients and long‐range cell‐to‐cell communications, incorporating PDEs and ODEs into the models is necessary to represent the distribution of diffusive molecules.

Continuum models are often employed for systems with scales ranging from millimeters to micrometers, where individual cell dynamics might be less relevant. Instead, these models use cell density or order parameters to represent groups of cells. For example, continuum models are commonly used to study colony expansion mechanisms based on cell diffusion and movement, biofilm formation, and the hydrodynamics of colonies.

## 5 SUMMARY AND CONCLUSION

We reviewed the multifaceted aspects of bacterial colony development, encompassing physiological intricacies, social interactions, innovative experimental technologies, and modeling approaches. Our review underscored how bacterial colonies exhibit complex behaviors and organizational structures that profoundly impact their survival and functionality.

The integration of advanced technologies, including fluorescence‐based microscopy, flow cytometry, and spatially resolved transcriptomics, has revolutionized our ability to visualize and quantify subpopulations and gradients within colony structures. These methodologies have extended the field of view from 2D to spatial–temporal 3D bacterial communities, providing unprecedented insights into the spatial heterogeneity and temporal dynamics of bacterial communities. They have revealed intricate communication mechanisms, metabolic cross‐feeding, and cooperative behaviors.

Mathematical modeling has emerged as a crucial complement to experimental approaches, offering a framework to understand the complex, nonlinear dynamics of colony formation. From reaction–diffusion equations to discrete element models, these theoretical tools have elucidated key drivers of pattern formation and colony expansion, bridging the gap between molecular‐level interactions and macroscopic colony behaviors.

With these advances in recent years, new questions have arisen that we believe are important for understanding bacterial communities. First, current research focuses on patterns at the 2D level, assuming that the scales in the height direction are much shorter than those in the *X*‐ and *Y*‐ directions, and there is a lack of methods to precisely quantify cells in the height direction. Recent works have revealed heterogeneity in metabolism within a simple colony developed from a single cell [54, 66]. It is necessary to focus more on the spatiotemporal dynamics of colony formation in 3D, despite significant challenges in both experimental technology and mathematical modeling. Secondly, the self‐organization of metabolic interactions in bacterial communities and their consequences in macroscopic patterns remain elusive. Thirdly, current works are based on laboratory‐confined conditions. However, biofilms in natural systems encounter fluctuations in nutrients and physical obstacles. Understanding these factors in colony development requires more efforts in quantitative methods at different scales to bridge the gaps between homogeneous laboratory conditions and fluctuating environments in the real world.

## AUTHOR CONTRIBUTIONS

**Jingwen Zhu**: Conceptualization; funding acquisition; investigation; writing—original draft. **Pan Chu**: Conceptualization; investigation; writing—original draft. **Xiongfei Fu**: Conceptualization; funding acquisition; project administration; writing—review & editing.

## CONFLICT OF INTEREST STATEMENT

The authors declare no conflicts of interest.

## ETHICS STATEMENT

This article does not contain any studies with human or animal materials performed by any of the authors.

## Data Availability

The data that support the findings of this study are available from the corresponding author upon reasonable request.
